# Global urban expansion offsets climate-driven increases in terrestrial net primary productivity

**DOI:** 10.1038/s41467-019-13462-1

**Published:** 2019-12-05

**Authors:** Xiaoping Liu, Fengsong Pei, Youyue Wen, Xia Li, Shaojian Wang, Changjiang Wu, Yiling Cai, Jianguo Wu, Jun Chen, Kuishuang Feng, Junguo Liu, Klaus Hubacek, Steven J. Davis, Wenping Yuan, Le Yu, Zhu Liu

**Affiliations:** 1 School of Geography and Planning, Sun Yat-sen University, 135 West Xingang RD., Guangzhou, 510275 China; 2 School of Geography, Geomatics, and Planning, Jiangsu Normal University, 101 Shanghai RD., Tongshan New District, Xuzhou, 221116 China; 3 School of Geographic Sciences, East China Normal University, 500 Dongchuan Road, Shanghai, 200241 China; 4 Department of Urban Studies and Planning, Massachusetts Institute of Technology, Cambridge, MA 02138 USA; 5 School of Life Sciences & School of Sustainability, Global Institute of Sustainability, Arizona State University, 427 East Tyler Mall, Tempe, AZ 85287 USA; 6 National Geomatics Center of China, 28 Lianhuachi West Road, Haidian District, Beijing, 100830 China; 7 Department of Geographical Sciences, University of Maryland, 2181 Samuel J. LeFrak Hall, 7251 Preinkert Drive, College Park, MD 20742 USA; 8 School of Environmental Science and Engineering, Southern University of Science and Technology, Shenzhen, 518055 China; 9 Center for Energy and Environmental Sciences (IVEM), Energy and Sustainability Research Institute Groningen (ESRIG), University of Groningen, Groningen, 9747 AG Netherlands; 10 Department of Environmental Studies, Masaryk University, Jostova, 10, 602 00 Czech Republic; 11 International Institute for Applied Systems Analysis, Schlossplatz 1, A-2361 Laxenburg, Austria; 12 Department of Earth System Science, University of California, Irvine, 3232 Croul Hall, Irvine, CA 92697-3100 USA; 13 Department of Earth System Science, Tsinghua University, Beijing, China

**Keywords:** Climate-change mitigation, Environmental impact, Carbon and energy, Interdisciplinary studies

## Abstract

The global urbanization rate is accelerating; however, data limitations have far prevented robust estimations of either global urban expansion or its effects on terrestrial net primary productivity (NPP). Here, using a high resolution dataset of global land use/cover (GlobeLand30), we show that global urban areas expanded by an average of 5694 km^2^ per year between 2000 and 2010. The rapid urban expansion in the past decade has in turn reduced global terrestrial NPP, with a net loss of 22.4 Tg Carbon per year (Tg C year^−1^). Although small compared to total terrestrial NPP and fossil fuel carbon emissions worldwide, the urbanization-induced decrease in NPP offset 30% of the climate-driven increase (73.6 Tg C year^−1^) over the same period. Our findings highlight the urgent need for global strategies to address urban expansion, enhance natural carbon sinks, and increase agricultural productivity.

## Abstract (web summary)

Robust estimates of either urban expansion worldwide or the effects of such phenomenon on terrestrial net primary productivity (NPP) are lacking. Here the authors used the new dataset of global land use to show that the global urban areas expanded largely between 2000 and 2010, which in turn reduced terrestrial NPP globally.

## Introduction

The terrestrial biosphere is a large carbon sink in the global carbon cycle, with a net carbon uptake of 1–4 Pg C year^−1^ during the last few decades^1,2^. As the initial production stage in which atmospheric carbon dioxide (CO_2_) is fixed by plants, terrestrial net primary productivity (NPP) increased by ~190 Tg C year^−1^ between 1982 and 1999^3^. The NPP increase occurred despite the projected decrease in terrestrial carbon uptake from climate-carbon cycle simulations^4,5^. However, temporal trends of global terrestrial NPP since 2000, as well as its underlying mechanics, remain uncertain in the context of climate variability and anthropogenic disturbance (e.g., urban expansion and deforestation)^6–8^. As one of the important components of human-associated disturbance, urban land expansion and its effects on terrestrial NPP have been widely examined using a case-based approach^9–12^. However, these interactions cannot be accurately estimated at a global scale due to the lack of reliable global land-use data with a high spatiotemporal resolution^13,14^. Furthermore, at the global scale, it is difficult to clarify the relative roles of climate variability and urban land expansion on terrestrial NPP^15,16^.

GlobeLand30, one of the newly developed dataset with a 30-m resolution, has the advantages of both spatiotemporal detail and data accuracy (see Methods)^17,18^. Here, we use this dataset to analyze the change in global urban lands from 2000 to 2010 in much greater detail than has previously been possible. Here, the urban lands mainly refer to the area modified by human activities, including all kinds of habitation, industrial and mining area, transportation facilities, interior urban green zones and water bodies^18^. We then estimated global terrestrial NPP using the Moderate Resolution Imaging Spectrometer (MODIS) NPP dataset (MOD17A3), the Carnegie–Ames–Stanford Approach (CASA)^19,20^ and one of the Lund-Potsdam-Jena Dynamic Global Vegetation Model (LPJ-Hydrology)^21^. On this basis, change in the global terrestrial NPP was further analyzed using the approach of multi-model ensemble mean (MMEM). To isolate the effects of climate variability and urban expansion from other mechanisms (e.g., CO_2_ fertilization, nitrogen deposition, and wildfire), we estimated terrestrial NPP as ‘urban-expansion-based NPP’ and ‘climate-variability-based NPP’ in two separate simulations, in which either climate drivers or urban land areas were held constant. Specifically, urban-expansion-based NPP was simulated by holding fixed climate drivers and varied urban land use. Conversely, climate-variability-based NPP was calculated using a fixed urban land distribution (i.e., the 2000 state) and a varied climate driver from 2000 to 2010. Further details of our approach are provided in Methods. As results, we found that global urban expansion remarkably reduced the terrestrial NPP over the period 2000–2010 (22.4 Tg C year^−1^), which offset 30% of the climate-variability-driven NPP increase. In doing so we provide a comprehensive assessment of urbanization-induced NPP change in comparison with climate-variability-driven change.

## Results

### Global urban expansion

**Fig. 1:**
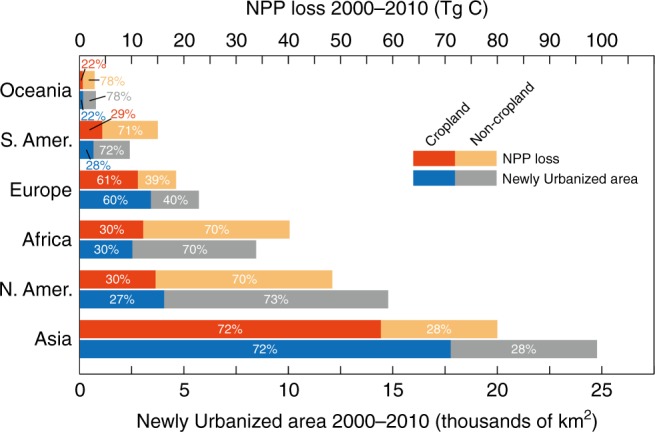
Continental summaries for all newly urbanized areas (which were converted from both non-cropland and cropland), cropland-converted newly urbanized area (which was converted from cropland), and their corresponding NPP losses between 2000 and 2010. The NPP losses from both newly urbanized land and cropland-converted newly urbanized area were calculated for Asia, North America (N. Amer.), Africa, Europe, South America (S. Amer.) and Oceania based on the urban-expansion-based NPP and global urban land area in 2000 and 2010. NPP denotes net primary productivity.

We found that global urban lands expanded much faster than expected between 2000 and 2010^22^. Specifically, the world’s urban area increased by 5694 km^2^ per year over this 10-year interval, which represents ~5% of total urban area in 2000. As projected, there were big differences among urban expansion area on each continent (Supplementary Figs. 1 and 2), with the largest urban land expansion occurring in Asia and North America (Fig. 1). In particular, urban expansion in Asia was marked, involving a total area of 24,770 km^2^. In addition, 14,780 km^2^ was converted to urban land use in North America. Together, 69% of the global urban expansion took place in these two continents between 2000 and 2010. In addition, urban land use in Africa expanded quickly during this period—faster than in other continents. Although the newly urbanized land in Africa covered only 8462 km^2^, this urban expansion accounted for ~12% of the total urban area of the continent in 2000. This could be associated with the underlying driver such as population growth. However, the outcome of the urban expansion in Africa in terms of economic growth was poor^23^. The smallest increase in urban area occurred in Europe, which is already highly urbanized. These geographically divergent results are likely associated with national and regional differences in socio-economic and political conditions^22^. In particular, urban expansion in China was 16,053 km^2^ between 2000 and 2010, ~65% of total urban land growth in Asia and 28% of the global urban expansion (Supplementary Table 1). Despite a relatively low rate of urban land growth in the United States, newly urbanized areas here (11,773 km^2^) accounted for 21% of the worldwide total (Supplementary Table 1; Supplementary Fig. 1).

### The impact of global urban expansion on terrestrial NPP

Although only occupying a small proportion of the earth’s land surface (0.04%), newly urbanized areas in the period 2000–2010 had a disproportionately effect on global terrestrial NPP. Specifically, global urban expansion was associated with a 22.4 Tg C year^−1^ reduction in the NPP, which is ~9% of the carbon emissions from fossil fuel and cement emissions worldwide^24^. In particular, China and United States experienced considerable NPP reductions of 40.6 Tg C and 47.5 Tg C, respectively (see Supplementary Note 1; Supplementary Table 1). In the United States, rate of the NPP losses in the newly urbanized area between 2000 and 2010 could be doubled compared to that of the entire urbanized area in this country in 1994–1995 (41.5 Tg C year^−1^)^10^, assuming same NPP performance (and thus NPP loss rate) of all urban lands with the urban expansion area between 2000 and 2010. In addition, prior research found that urban land development not only reduced terrestrial NPP, but also resulted in the loss of a significant amount of fertile agricultural soil^9,25,26^. However, it is not currently certain whether this phenomenon exists at the global scale. Figure 1 compares global urban expansion and the corresponding changes the in NPP for different continents between 2000 and 2010. The proportion of these changes associated with the conversion of cropland and non-cropland (e.g., unmanaged land) to urban use are also shown. Our results show that newly urbanized area had a disproportionately high impact on terrestrial NPP in South America and Africa (Fig. 1). This phenomenon could be associated with the large conversion of highly productive ecosystems such as tropical rainforest to urban land use in these two continents. In addition, such conversions raise additional concerns. For instance, urban land development was found to associate with cropland losses, indicating an irreversible loss of agricultural capacity. As shown in Fig. 1, 72% of Asia’s urban land growth involved encroachment on cropland. In North America, although only 27% of the newly urbanized area (4060 km^2^) was converted from cropland, it is approximately twice the area of converted cropland in Africa (2536 km^2^).

**Fig. 2:**
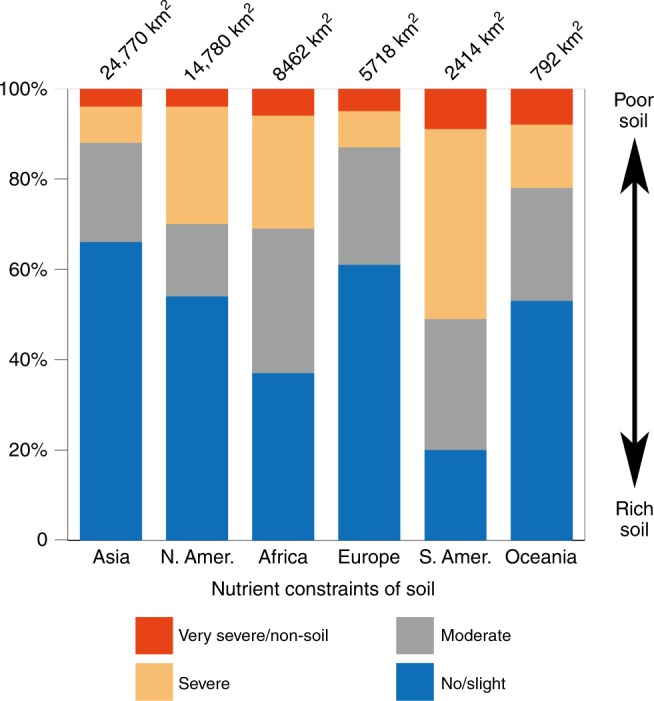
Soil nutrient constraint in newly urbanized lands (2000-2010) by continents. The constraint is grouped into the following four categories based on reference ^27^: No or slight constraint, moderate constraint, severe constraint, and very severe constraint or non-soil.

Figure 2 highlights another dimension of the effects of global urban expansion on agricultural capacity, with more than 50% of newly urbanized areas occurring on highly fertile soils (i.e., no or slight nutrient constraint in Fig. 2) in all continents except Africa and South America^27^. In Africa, the proportion was only 37% probably owing to human-induced soil nutrient depletion^28,29^. In addition, the low proportion for South America could be associated with the widespread distribution of low and medium quality soil across the continent^30^. Given the ever-growing human population and looming impacts of climate variability on agricultural yields^31^, this sacrifice of highly fertile land for urban growth has potentially significant implications for terrestrial NPP and even crop production worldwide.

### Effect of urban expansion and climate variability on the NPP

**Fig. 3:**
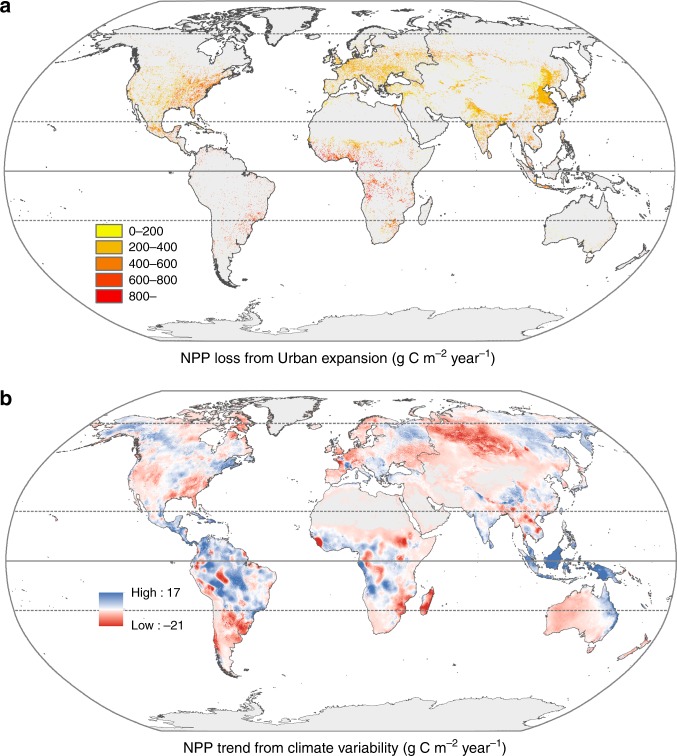
Spatiotemporal trends of the NPP caused by urban expansion **a** and climate variability **b** between 2000 and 2010. This figure reflects the spatiotemporal trends of the global terrestrial NPP caused by urban expansion and climate variability. To account for the two factors, two independent experiments (i.e., climate-variability-driven and urban-expansion-driven simulations) were performed to estimate the terrestrial NPP as ‘urban-expansion-based NPP’ and ‘climate-variability-based NPP’, in which either climate drivers or urban lands were held constant. The trends of the urban-expansion-based NPP and the climate-variability-based NPP were then analyzed using linear regression analyses with time (i.e., year) as independent variable and corresponding NPP as dependent variable. NPP denotes net primary productivity.

We further compared the effects of urban expansion and climate variability on the terrestrial NPP for the period 2000–2010. The results show that the reduction in NPP associated with urban expansion offset a substantial fraction (30%) of the increase in the NPP resulting from climate variability over the same period (equating to 73.6 Tg C year^−1^). Figure 3 provides spatial details of the effects of urban expansion (Fig. 3a) and climate variability (Fig. 3b) on the terrestrial NPP between 2000 and 2010. The effect of urban expansion was much more concentrated, accounting for a NPP reduction of up to 1123 g C m^−2^ year^−1^, as compared to the maximum reduction of ~21 g C m^−2^ year^−1^ due to climate variability. The NPP losses from urban expansion were mainly clustered in the temperate zones of eastern China, northern Indian subcontinent, eastern North America, and Europe (Fig. 3a). In addition, climate drivers played a critical role on changes in the terrestrial NPP in the study period (see Methods). For instance, changes in climate-variability-driven NPP were widespread with the exception of desert area, with substantial decrease occurring in the Amazon and other tropical regions (Fig. 3b) due to constrained solar radiation from persistent cloud cover and the stress of warming temperature (Supplementary Fig. 11; cf. ref. ^6^). We also found the greatest climate-variability-induced increases in the NPP at the temperate and high latitudes of the Northern Hemisphere, where warming lengthened the growing season (Fig. 3; Supplementary Fig. 11)^6,32^. These findings represent a continuation of the trends observed during the preceding years^3^.

Combining climate variability, urban expansion and residual factor (i.e., all other factors unexplained by climate variability and urban expansion, such as wildfire and nitrogen deposition), annual increase in global terrestrial NPP between 2000 and 2010 was ~97.4 Tg C year^−1^, which is ~49% less than between 1982 and 1999^3^. Spatially, our results show large NPP decreases in the Northern Hemisphere in 2001, with more persistent decreases in the Southern Hemisphere between 2002 and 2010 (Supplementary Fig. 4).

**Fig. 4:**
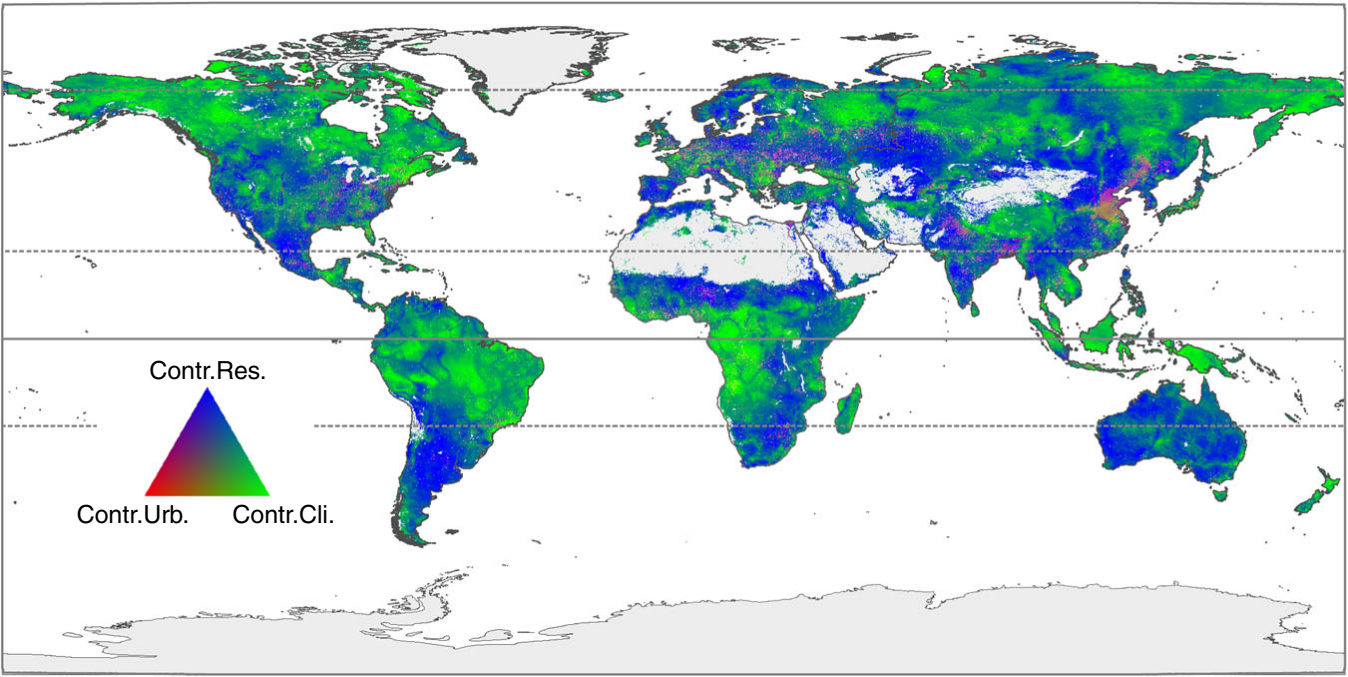
Relative contributions of urban expansion (Contr.Urb.), climate variability (Contr.Cli.) and residual factor (Contr.Res.) to change in terrestrial NPP from 2000 to 2010. This figure mainly describes spatial pattern of the relative role of climate effect, human activity and unexplained factors on terrestrial NPP change. By conducting two independent experiments as climate-variability-driven and urban-expansion-driven simulations, global terrestrial NPP was estimated as ‘urban-expansion-based NPP’ and ‘climate-variability-based NPP’. The residual factor, which is not explained by urban expansion and climate variability, was also analyzed. Relative contributions of urban expansion, climate variability, and residual factor were determined as the proportions between the trends of climate-variability-based NPP, urban-expansion-based NPP, residual factor and the sum of their absolute values. The spatial explicit approach partitioning the relative influences of urban expansion, climate variability and residual factor provides insight into the mechanisms of terrestrial ecosystem change (see Methods). NPP denotes net primary productivity.

Figure 4 shows the relative contributions of urban expansion (red), climate variability (green), and residual factor (blue) to changes in the terrestrial NPP (calculated as the cumulative effect of each component relative to the total NPP change due to all factors) at the cell scale from 2000 to 2010 worldwide. Overall, the contribution of urban expansion was evident in newly urbanized areas such as Asia, North America and Europe, accounting for an average of 71% of the total NPP change during 2000–2010 (red areas in Fig. 4; Supplementary Fig. 5). In contrast, climate variability accounted for 37% of the total change in terrestrial NPP (green areas in Fig. 4; Supplementary Fig. 3). However, residual factor dominated in many areas, explaining an average of 62% of the total NPP change. It played a vital role in the terrestrial NPP in Australia, Africa, American southwest, and Indian subcontinent (blue areas in Fig. 4; Supplementary Fig. 6). This could be associated with the changes in atmosphic CO_2_ concertration (Supplementary Fig. 7). For instance, CO_2_ fertilization on vegetation growth was frequently observed, especially in water-limited regions^3,33,34^. In addition, wild fire could be attributed to the variation of terrestrial NPP, and acted as a prevalent driver of boreal carbon balance in northern Russia and southern Canada^35^.

## Discussion

The GlobeLand30 was proved to be accurate and reliable to capture global urban land changes, with a precision ten times greater than most of previous land use/cover datasets (e.g., the University of Maryland land-cover map and MODIS land-cover maps)^17,18^. Based on this dataset, our result shows that global urban land expanded faster than expected between 2000 and 2010. The expansion rate was approximately three times that of the global urban expansion during the previous 30-years between 1970 and 2000 according to the meta-analysis of 326 studies^22^. In addition, we found that 44.6% of the urban expansion involved an encroachment onto cropland. Considering increasing losses in the cropland predicted by 2030, an effective regulation of urban expansion is urgently needed to promote crop production worldwide^36^.

Urban expansion not only substantially transforms the landscape, but also alters biogeochemical cycle and the photosynthetic productivity of terrestrial ecosystems. Past studies have shown that urban expansion reduced terrestrial NPP at the city to country scale due to the replacement of natural vegetation into impervious surfaces^10,37,38^. Despite this, however, vegetation growth can be enhanced in urban environment^12,39^. This enhancement can be associated with the introduction of highly productive vegetation, as well as heat-island effect in urban area^12,40^. Nevertheless, such growth enhancement is likely insufficient to offset the direct loss of productivity caused by the replacement of productive vegetation with impervious surfaces. Furthermore, we found that global urban expansion reduced global terrestrial NPP from 2000 to 2010 by an amount approximately equal to 9% of worldwide carbon emissions from fossil fuels and cement production. Considering the projected increases in urban land use^36^ and fossil-fuel-consumption emissions^41^, additional research and policy are needed to regulate urban land growth at both national and international levels^42,43^. Furthermore, we note that global urban expansion has already caused large losses in fertile lands. The effects of the increases in cropland productivity that can be achieved via climate variability adaption strategies (i.e., changing planting dates, new irrigation regimes, and improved residue management)^44^ may be largely limited by the fast urban expansion at a global scale.

As for the effects of climate variability on the terrestrial NPP, past studies have consistently shown a climate-variability-induced increase for the period 1982–1999^3,45,46^. However, in comparison, there is a lack of consensus on the NPP trend since 2000. Zhao and Running^6^ reported a drought-induced reduction in global terrestrial NPP of 0.55 Pg C from 2000 to 2009^6^. However, Medlyn^47^ and Samanta et al.^48^ argued that this estimate could be biased by temperature dependence, gap-filling of satellite data, and generally weak correlations with field observations^6,47,48^. In addition, Potter et al.^7^ estimated that global terrestrial NPP increased by 0.14 Pg C during the same period^7^. The confliction could be associated with the different approaches they employed to model the effects of environmental stress on terrestrial NPP^6,7^. Our findings partly agree with ref. ^7^, reporting an increase in the NPP during 2000–2010. Furthermore, spatial pattern of the NPP anomalies in our analysis was similar to that reported by refs. ^6,7^.

Despite the widely documented evidence of individual effects of urban expansion and climate variability on the terrestrial NPP, little is known about their interactive effects when integrating the two factors as a whole, especially at a global scale^16^. We found that the NPP reduction associated with urban expansion (22.4 Tg C year^−1^) offset a substantial fraction (30%) of the increase in the NPP due to climate variability (73.6 Tg C year^−1^) during the period 2000–2010. In particular, the contribution of urban expansion was even more pronounced in newly urbanized areas, accounting for an average of 71% of the total NPP change in the study period. This reveals that, at a global scale, urban expansion was the main driving force behind terrestrial NPP changes in newly urbanized areas. Similar results have also been reported previously^15,16^. In addition, climate variability accounted for 37% of the total change in the terrestrial NPP during this period. Factors other than climate variability and urban expansion explained an average of 62% of the total NPP change in some regions, including the boreal region of northern Russia and Asia. These results are partly in agreement with ref. ^15^, which reported a dominant control of climate variability and residual factors on the terrestrial NPP in the areas with natural vegetation.

Together, our work provides a comprehensive assessment of the remarkable effects of global urban expansion on the terrestrial NPP in comparison with climate-variability-driven changes. Despite it, multiple uncertainties still remain in our results when separating the effects of urban expansion and climate variability on the terrestrial NPP. For instances, urban landscapes usually include various land cover types such as trees, lawns, and impervious surfaces, all of which exhibit different levels of productivity. It is particularly difficult to simulate urban NPP accurately without complex experimental designs. Besides addressing land cover types, other biological, chemical, and physical factors, including topography, air pollutants, and nitrogen deposition should be given further consideration^6,40,49^. In addition, while previous studies have mostly focused on land use/cover changes related to natural vegetation, in this context, carbon cycle models should incorporate urban land development as one of their critical components^50^.

## Methods

### Study design

In recent decades, several global land-cover maps have been developed using satellite data at moderate to coarse resolutions (i.e., from 250 to 1000 m), including the University of Maryland land-cover map, the MODIS land-cover maps, and the GlobCover land-cover maps. Because of the mismatch between fine urban expansion and coarse land-cover maps, it is difficult to derive accurate urban land use change using these maps. Reference^14^ developed the first 30-m global land-cover dataset (FROM-GLC) using the satellite-based data from Landsat Thematic Mapper (TM) and Enhanced Thematic Mapper Plus (ETM+). However, its usefulness in deriving global urban expansion is limited because of inadequate temporal coverage. The GlobeLand30 dataset, which covers the years 2000 and 2010 at a 30-m resolution, has advantages on both spatiotemporal details and data accuracy^17,18^. In past studies, the GlobeLand30 dataset was evaluated through sample-based validation or comparison with existing land-cover products by third-party researchers from more than 10 countries^18^. Here, accuracy of the GlobeLand30 dataset was further evaluated by comparing it to the Global Human Built-up And Settlement Extent (HBASE) dataset using the method proposed by refs. ^51,52^. According to the box-plot analysis of the kappa coefficient, the GlobeLand30 dataset has an overall higher accuracy at fifteen urban ecoregions than the HBASE dataset (Supplementary Figs. 8 and 9).

To effectively capture the detail distribution of urban land use/cover, the GlobeLand30 dataset was employed to detect global urban expansion from 2000 to 2010. In addition, global terrestrial NPP was estimated over the period 2001–2010 using the MODIS NPP dataset (i.e., MOD17A3) from the MODIS gross and net primary productivity algorithms (MOD17A2/A3)^53^ and the results from two ecosystem models (i.e., the CASA and the LPJ-Hydrology). On this basis, changes in the global terrestrial NPP were estimated by using the approach of multi-model ensemble mean (MMEM). To isolate the effects of climate variability and urban expansion from other mechanisms, two separate experiments were conducted—an ‘urban-expansion-driven’ simulation and a ‘climate-variability-driven’ simulation, respectively. Urban-expansion-based NPP was then estimated by holding climate drivers fixed and varying urban lands from 2000 to 2010. In contrast, climate-variability-based NPP was estimated by holding urban lands fixed (at its 2000 level) and varying climate drivers from 2000 to 2010. The relative contributions of urban expansion/climate variability/residual factor were alternately examined as proportions between the trends of climate-variability-based NPP/urban-expansion-based NPP/residual factor and the sum of their absolute values.

### Ecosystem model analysis

We simulated global terrestrial NPP using the CASA and one of the Lund-Potsdam-Jena Dynamic Global Vegetation Model (LPJ-Hydrology)^19–21^. The CASA, a light use efficiency (LUE) model^54^, is widely used to estimate terrestrial NPP at regional to global scales^7,55,56^. According to this approach, the NPP (g C m^−2^) in a given location *x* and time *t* is calculated as follows:

1 $$NPP(x,t) = S(x,t) \times FPAR \times 0.5 \times T_1(x,t) \times T_2(x,t) \times W(x,t) \times \varepsilon _{\max }$$

where *S*(*x*, *t*) is solar surface irradiance, *MJ* *m*^*−2*^); *FPAR* is the fraction of photosynthetic active radiation absorbed by green vegetation, which is calculated using satellite-derived normalized differential vegetation index (NDVI) data; the factor 0.5 represents the fact that approximately half the incoming shortwave solar radiation is in the photosynthetic active radiation wavelength (0.4–0.7)^19^; and account for the effects of very high and very low temperature stress, respectively; *W* represents moisture stress factor; and *ε*_max_ is maximum LUE. The maximum LUE () is uncertain across land use/cover types, spatial scales and vegetation coverage^57,58^. Several methods are available to derive the *ε*_max_, including eddy covariance technology, productivity model inversion and quantum efficiency reckoning^59^. The modified least square algorithm, which was proposed by ref. ^57^, was proved to be effective in simulating the *ε*_max_^11,57^. According to this algorithm, the term *ε*_max_ was calibrated as:

2 $$E(x) = \mathop {\sum}\limits_{i = 1}^j {(m_i - n_ix)^2}$$

where *i* represents the samples for a specific land use/cover type; *j* is the number of samples; *m* represents field NPP values; *n* is the product of *S*(*x*, *t*), *FPAR*, *T*_1_, *T*_2_ and *W*_;_ *x* is the maximum LUE to be determined. By minimizing the errors between field-measured NPP (*m*) and the term *n*, *ε*_max_ was calibrated for each land use/cover type.

To fit the CASA model, land use/cover data were further compiled by combining the GlobeLand30 dataset with one of the MODIS product (i.e., MCD12Q1 dataset based on International Geosphere–Biosphere Programme (IGBP) classification scheme). That is, urban land cover was compiled from the GlobeLand30 dataset using Geographic Information System (GIS). In addition, natural vegetation including evergreen needleleaf forest (ENF), evergreen broadleaf forest (EBF), deciduous needleleaf forest (DNF), deciduous broadleaf forest (DBF), mixed forest (MF), shrub, savanna, and grassland, was compiled from the MCD12Q1 dataset. Based on this, the CASA model was calibrated using the algorithm of modified least squares with a total of 2843 field-based NPP records from the corrected Global Primary Production Data Initiative (GPPDI) dataset. A detailed description of the calibration steps can be found in refs. ^20,57^. The values of our simulated are listed in Supplementary Table 2. It varied from 0.389 g C MJ^−1^ for any other land use/cover type (e.g., wetland and cropland) to 0.660 g C MJ^−1^ for evergreen broadleaf forest (EBF). These values were mostly between 0.389 g C MJ^−1^ proposed in ref. ^19^ and the prescribed values employed when producing the MODIS NPP product (MOD17A2/A3)^53^. In addition, our calibrated *ε*_max_ values were similar to those proposed by other studies^57^.

To reduce model uncertainty when estimating the terrestrial NPP, LPJ-Hydrology, one of modified Lund-Potsdam-Jena Dynamic Global Vegetation Model, was also used to simulate the ecosystem dynamics^21^. The original Lund-Potsdam-Jena Dynamic Global Vegetation Model (LPJ-DGVM) simulates photosynthesis, respiration, fires, growth, and competition of different plant functional types (PFTs) through explicit representations of vegetation structure, dynamics, competition between PFT populations and soil biogeochemistry^60,61^. However, the LPJ-DGVM often requires a long spin-up simulation from bare soil to reach an equilibrium state of vegetation and soil carbon pools^21,61,62^. Reference^21^ proposed the LPJ-Hydrology, which incorporates satellite-derived land use/cover data into the LPJ-DGVM instead of dynamically simulating it. Here, the LPJ-Hydrology was conducted prescribing the land use/cover input at its 2006 state to simulate the terrestrial NPP. Following general protocol, we first span-up the LPJ-Hydrology for 1000 years to reach equilibrium state of the vegetation and soil pools^61^. The model was then continuously executed to estimate the terrestrial NPP using climate and CO_2_ data for the period 2000–2010 as inputs. To facilitate the comparability of the NPP results from the CASA and the LPJ-Hydrology, we kept the input data of both models the same or identical. Concretely, the CASA was driven by the data on temperature, precipitation, solar radiation and NDVI from 2000 to 2010. In addition, the LPJ-Hydrology was forced by temperature, precipitation, number of wet days, cloud cover and CO_2_ concentration over the same period. To validate the CASA and the LPJ-Hydrology, our estimated NPP results were compared with the field-based NPP records from the GPPDI dataset. Significant correlations were found between the field-based NPP and our simulated NPP from both the CASA (*R* = 0.963; *N* = 2843; *P* = 0.000) and the LPJ-Hydrology (*R* = 0.962; *N* = 2843; *P* = 0.000) (Supplementary Fig. 10). Furthermore, consistency was also found among the NPP dataset simulated using the CASA, our estimated NPP from the LPJ-Hydrology model, and the NPP from one of the MODIS product (MOD17A3). This indicates the reliability of the CASA and the LPJ-Hydrology when simulating the global terrestrial NPP.

Based on the NPP from the MOD17A3 dataset and the simulated NPP results from the CASA and the LPJ-Hydrology, global terrestrial NPP was estimated using the MMEM approach. Anomalies of global terrestrial NPP were then analyzed by subtracting mean NPP over past 10 years (2000–2010) from annual terrestrial NPP, respectively. We found that increases in the terrestrial NPP occurred over large areas of North America, Western Europe and Eastern Asia (Supplementary Fig. 4). However, negative NPP anomalies were also found in the Northern Hemisphere in 2001 and in the Southern Hemisphere for most of the study period (Supplementary Fig. 4).

### Isolate the impact of urban expansion and climate on the NPP

To examine the effects of climate-related drivers on global terrestrial NPP, we performed correlation analyses in both hemispheres and for the entire globe between our estimated terrestrial NPP and some other factors, including temperature, precipitation and solar radiation. Significant correlations were found between the terrestrial NPP and these climate drivers at the global scale (Supplementary Table 3). Spatially, the observed NPP increases at northern middle and high latitudes were largely consistent with rising temperature (Supplementary Fig. 11a, b). These results indicate the critical impacts of climate-related drivers on the change in terrestrial NPP.

To ensure consistency with the interactive framework between urban ecosystems and environmental changes^63^, global terrestrial NPP was analyzed in two independent experiments, specifically using a climate-variability-driven simulation and an urban-expansion-driven simulation. In the climate-variability-driven simulation, we calculated climate-variability-based NPP by prescribing changing climate drivers (i.e., monthly temperature and monthly precipitation from 2000 to 2010) and fixed urban land distribution at its 2000 state. That is, the CASA was driven by varied observations of temperature and precipitation from 2000 to 2010, with other drivers unchanged (e.g., averaged solar radiation, averaged NDVI and fixed urban land distribution in 2000). Similarly, the LPJ-Hydrology was forced by varied observations of temperature and precipitation from 2000 to 2010 and some other averaged observations including number of wet days, cloud cover and atmospheric CO_2_ concentration over the same period. To reflect natural land cover in pre-urban condition, pre-urban NPP in newly urbanized area during 2000–2010 was obtained using a neighborhood proxy method^10,11^. The pre-urban condition was defined as a condition of the original vegetation distribution prior to urban land development. Therefore, the pre-urban NPP represents the NPP in the absence of urban land development. We assumed that nearby non-urban land surfaces are the best proxy for urban lands before it is transformed. That is, the pre-urban NPP in urban cells was calculated by replacing their current NPP (post-urban NPP) with the mean post-urban NPP of nearby non-urban cells that corresponded to their original natural vegetation^10,11^. On this basis, the climate-variability-based NPP was compiled by aggregating pre-urban NPP in urban areas and the NPP results from the climate-variability-driven simulation for non-urban area.

In the urban-expansion-driven simulation, urban-expansion-based NPP was projected using changing urban land use/cover for 2000–2010 and constant climate drivers (e.g., a constant NPP distribution derived from the mean terrestrial NPP for the whole study period). The pre-urban NPP in the newly urbanized areas from 2000 to 2010 were first estimated using a neighborhood proxy method. By assuming urban lands as a carbon source (i.e., post-urban NPP (*NPP*_2010_) is zero), NPP variation caused by urban expansion was calculated for the newly urbanized areas (2000–2010) as negative pre-urban NPP (*NPP*_2000_) (i.e., *NPP*_2010_-*NPP*_2000_ = −*NPP*_2000_).

### Contribution of climate and urban expansion to NPP change

To analyze the relative contribution of urban expansion/climate variability/residual factor on the change of the terrestrial NPP, the trends of actual/climate-variability-based NPP were calculated using linear regression analyses with time (i.e., year) as the independent variable and corresponding NPP data as the dependent variable. Thus, the trends of the climate-variability-based (Δ*NPP*_climate-driver-based_) and actual NPP (Δ*NPP*_actual_) were calculated based on the following Equation:

3 $$y = \beta _0 + \beta _1t + \varepsilon$$

where *y* is the climate-variability-based/actual terrestrial NPP (g C m^−2^ year^−1^); *t* is the corresponding time (year); *β*_0_ is the intercept; *β*_1_ reflects the trend of the climate-driven/actual terrestrial NPP; and *ε* is the residual error.

Similarly, the trend of urban-expansion-based NPP (Δ*NPP*_urban-expansion-based_) was also calculated as the slope of urban-expansion-based NPP versus the corresponding time, by assuming a fixed rate of urban land expansion over the period 2000–2010. In addition, the trend of residual factor (ΔRes), which was not explained by urban expansion and climate variability, was calculated as the difference between the estimated actual NPP trend (Δ*NPP*_actual_) and the sum of the trends of climate-variability-based (Δ*NPP*_climate-driver-based_) and urban-expansion-based NPP (Δ*NPP*_urban-expansion-based_).

As shown in Eqs. 4–6, percent contributions of urban expansion (*Contr.Urb*), climate variability (*Contr*.*Clim*), and residual factor (*Contr*.Re*s*) were determined as proportions between the trends of climate-variability-based NPP, urban-expansion-based NPP, residual factor and the sum of their absolute values (Supplementary Figs 3. 5 and 6).

4 $$Contr.Urb = \frac{{\left| {\Delta Urb} \right|}}{{\left| {\Delta C\lim } \right| + \left| {\Delta Urb} \right| + \left| {\Delta \,{\mathrm{Re}} s} \right|}} \times 100\%$$

5 $$Contr.C\lim = \frac{{\left| {\Delta C\lim } \right|}}{{\left| {\Delta C\lim } \right| + \left| {\Delta Urb} \right| + \left| {\Delta \,{\mathrm{Re}} s} \right|}} \times 100\%$$

6 $$Contr.\, {\mathrm{Re}} s = \frac{{\left| {\Delta \, {\mathrm{R}} s} \right|}}{{\left| {\Delta C\lim } \right| + \left| {\Delta Urb} \right| + \left| {\Delta \,{\mathrm{Re}} s} \right|}} \times 100\%$$

where Δ*Urb* represents the trend of urban-expansion-based terrestrial NPP; ΔClim defines the trend of climate-variability-based NPP; ΔRe*s* denotes the trend of residual factor. More details can be found in reference ^15^.

### Global land-use datasets

GlobeLand30 dataset for 2000 and 2010 was obtained from the National Geomatics Center of China (http://www.globallandcover.com/GLC30Download/index.aspx). The dataset was produced using more than 20,000 Landsat and Chinese HJ-1 satellite images with a resolution of 30-m^17,18^. Besides the GlobeLand30, one of the land cover dataset from MODIS products (i.e., the MCD12Q1) was also collected from the Earth Observing System (EOS) Data Gateway at the Land Processes Distributed Active Archive Center (https://lpdaac.usgs.gov/data/get-started-data/collection-overview/). Specifically, the MCD12Q1 dataset from IGBP classification scheme was obtained for the year 2006 to capture the details of natural vegetation distribution.

### Satellite datasets

Two satellite-based datasets were employed in this study, including NDVI dataset and NPP dataset. In details, the NDVI dataset and the NPP dataset were also downloaded from the EOS data gateway at the Land Processes Distributed Active Archive Center. To facilitate large-scale calculations, the NDVI and NPP datasets from MODIS products (i.e., MOD13A3 and MOD17A3) were obtained for 2000–2010 at 1-km resolution.

### Climate datasets

The climate datasets in this study include mean temperature, total precipitation, downward shortwave solar radiation, cloud cover, and wet-day frequency. Specifically, historical records of monthly temperature and total precipitation for 2000–2010 were retrieved from the Physical Sciences Division of the NOAA Earth System Research Laboratory (ESRL). Monthly solar radiation data were provided by the Terrestrial Hydrology Research Group at Princeton University (http://hydrology.princeton.edu/data.php). These datasets were produced by combining observation-based records with National Centers for Environmental Prediction/National Center for Atmospheric Research (NCEP/NCAR) reanalysis data^64^. In addition, monthly cloud cover and wet-day frequency data were obtained from the Climatic Research Unit (CRU), University of East Anglia (https://crudata.uea.ac.uk/cru/data/hrg/cru_ts_3.24.01/). To match the spatial resolution of the satellite-derived MODIS datasets (e.g., MOD17A3 and MOD13A3), all of the climate datasets were resampled to a resolution of 1-km although this does not increase the effective resolution of the data.

### Soil data

The soil data were obtained from the Harmonized World Soil Database (HWSD), which was produced via a collaboration among the Food and Agriculture Organization (FAO) of the United Nations, International Institute for Applied Systems Analysis (IIASA), ISRIC-World Soil Information, Chinese Academy of Sciences, and the Joint Research Centre of the European Commission (JRC)^65^. The HWSD is a raster dataset with a 30 arc-second resolution containing >16,000 different soil mapping units. The dataset assimilated existing regional and national updates of soil information (e.g., European Soil Database and 1:1,000,000 soil map of China) with 1:5 000 000 FAO-UNESCO Soil Map of the World (FAO, 1971–1981). In addition, dataset on global soil nutrients was also collected from ref. ^25^.

### Global terrestrial NPP and grain yield data

The global terrestrial NPP data in this study were compiled from corrected GPPDI dataset^66^. The GPPDI dataset covers 2523 individual sites and 5164 half-degree grid cells. It underwent extensive reviews under the Ecosystem Model-Data Intercomparison process. Here, the datasets were downloaded from Oak Ridge National Laboratory (http://daac.ornl.gov//NPP/guides/NPP_GPPDI.html). In addition, grain yield data in 2005 for 232 cities in China were compiled from statistical books, which was downloaded from China’s economic and social development statistics database (http://tongji.cnki.net/kns55).

### Global CO_2_ data

The CO_2_ dataset was obtained from the U.S. NOAA Earth System Research Laboratory (https://www.esrl.noaa.gov/gmd/ccgg/trends). Both CO_2_ and other greenhouse gases have been measured at a globally distributed network of air sampling sites for several decades^67^. On this basis, mean CO_2_ concentration was obtained to produce the dataset^68^.

The details of the aforementioned datasets are listed in Supplementary Table 4.

## Supplementary information

Supplementary Information

## Data Availability

The datasets in this study are available within this article and Supplementary Information files. All of them were obtained from publicly available data. The data which were derived from the original datasets but not aforementioned are available upon requests.
