# Supplementary material for: Global urban expansion offsets climate-driven increases in terrestrial net primary productivity
Source: Nat Commun. 2019 Dec 5;10:5558. doi: 10.1038/s41467-019-13462-1 (PMC6895113; doi:10.1038/s41467-019-13462-1)
Supplement: Supplementary file 1 — Supplementary Information [file 41467_2019_13462_MOESM1_ESM.doc]

SUPPLEMENATRY INFORMATION for

**Global urban expansion offsets climate-driven increases in terrestrial net primary productivity**

Liu et al.

**This file also includes:**

**Supplementary Figures**

Supplementary Figures 1 to 13

**Supplementary Tables**

Supplementary Table 1 to 4

**Supplementary Notes**

Supplementary Note 1

# Supplementary Figures


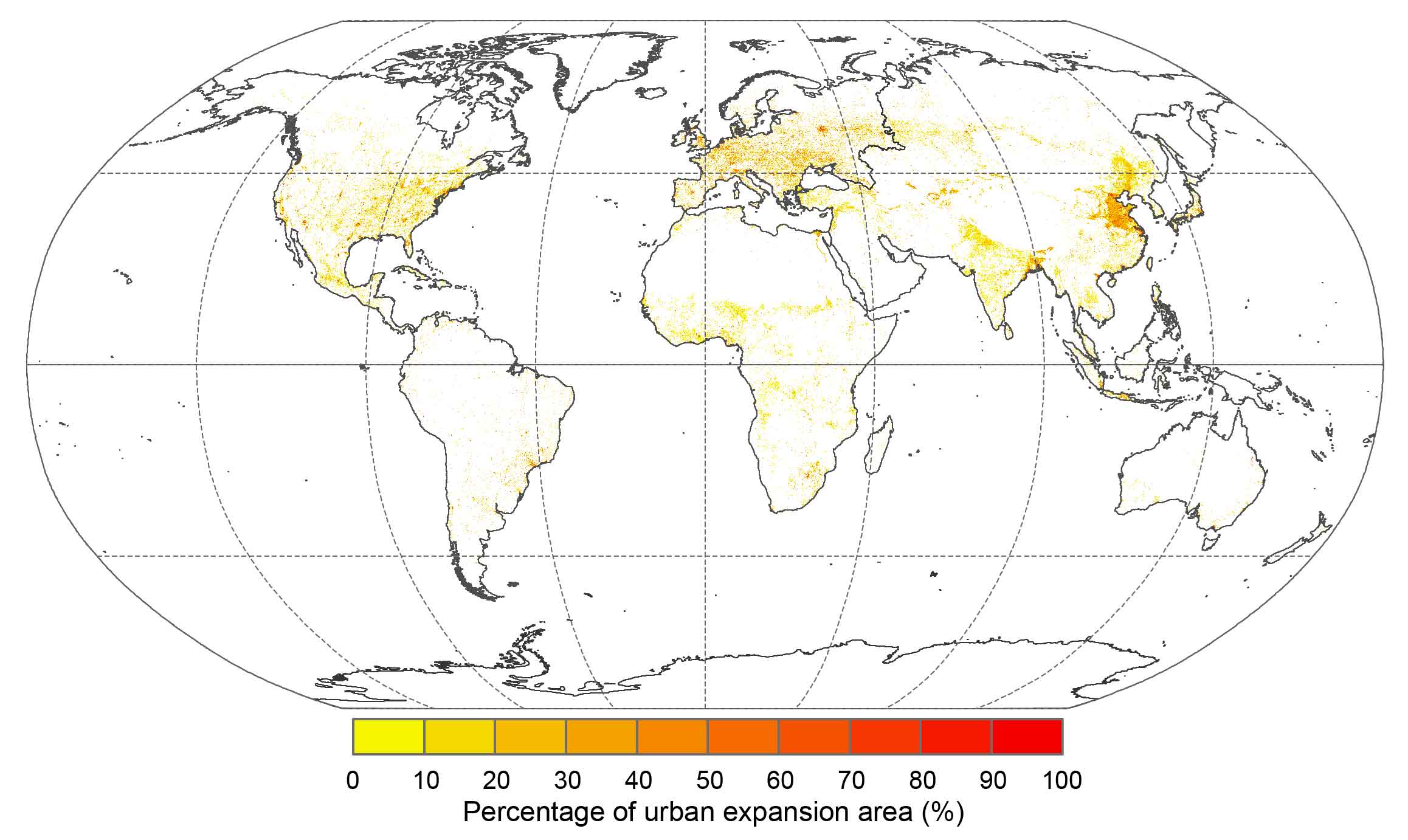


**Supplementary Figure 1. Percentage of urban expansion area during 2000**–**2010**. Here, urban lands were aggregated as fractional covers from 30m to 1000m for better visualization.





**Supplementary Figure 2. Urban land areas in 2000 and 2010, and its expansion percentage in Asia, Europe, Africa, South America (S. Amer.), Nouth America (N. Amer.) and Oceania.**


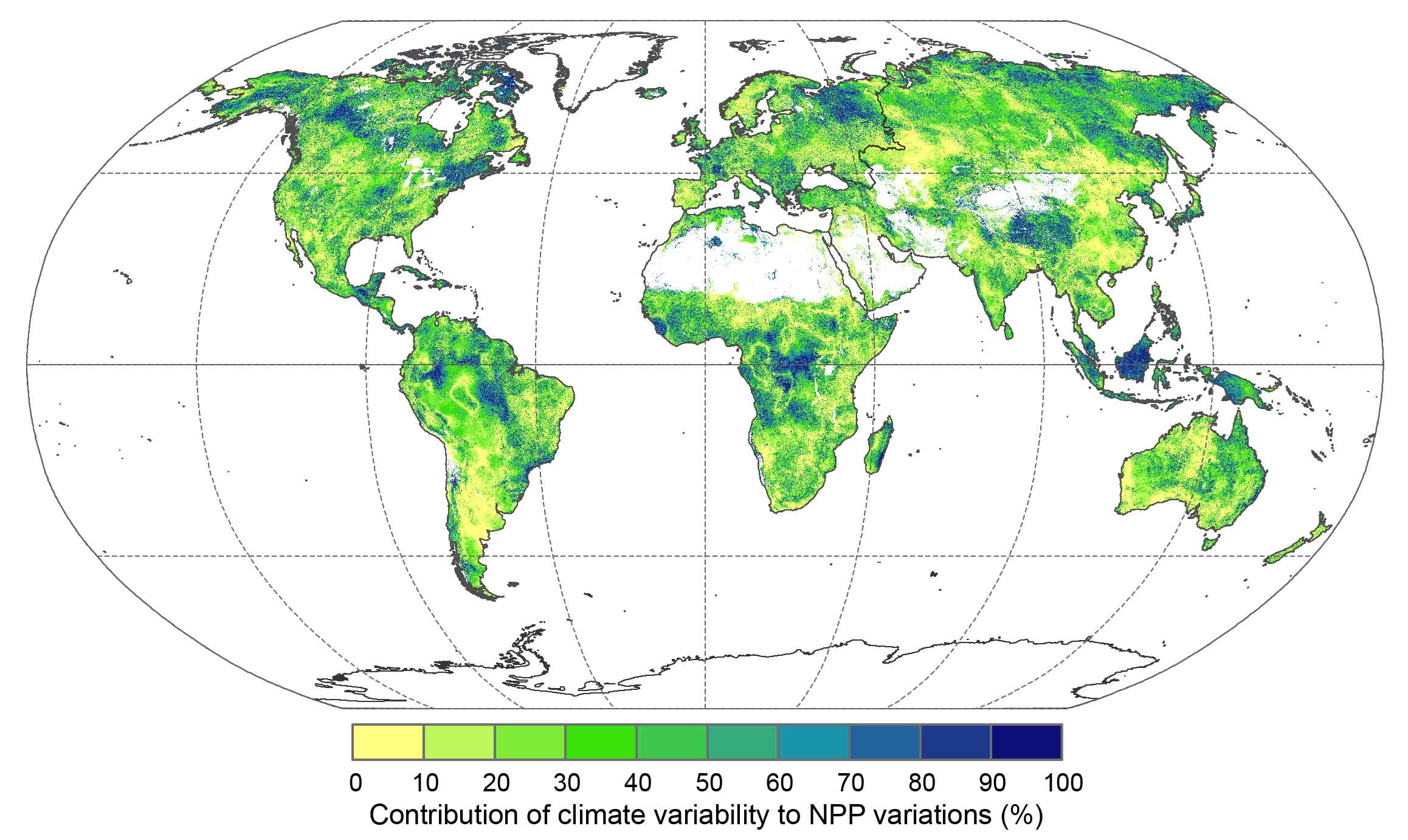


**Supplementary Figure 3. Contribution of climate variability to changes in terrestrial NPP from 2000 to 2010.**


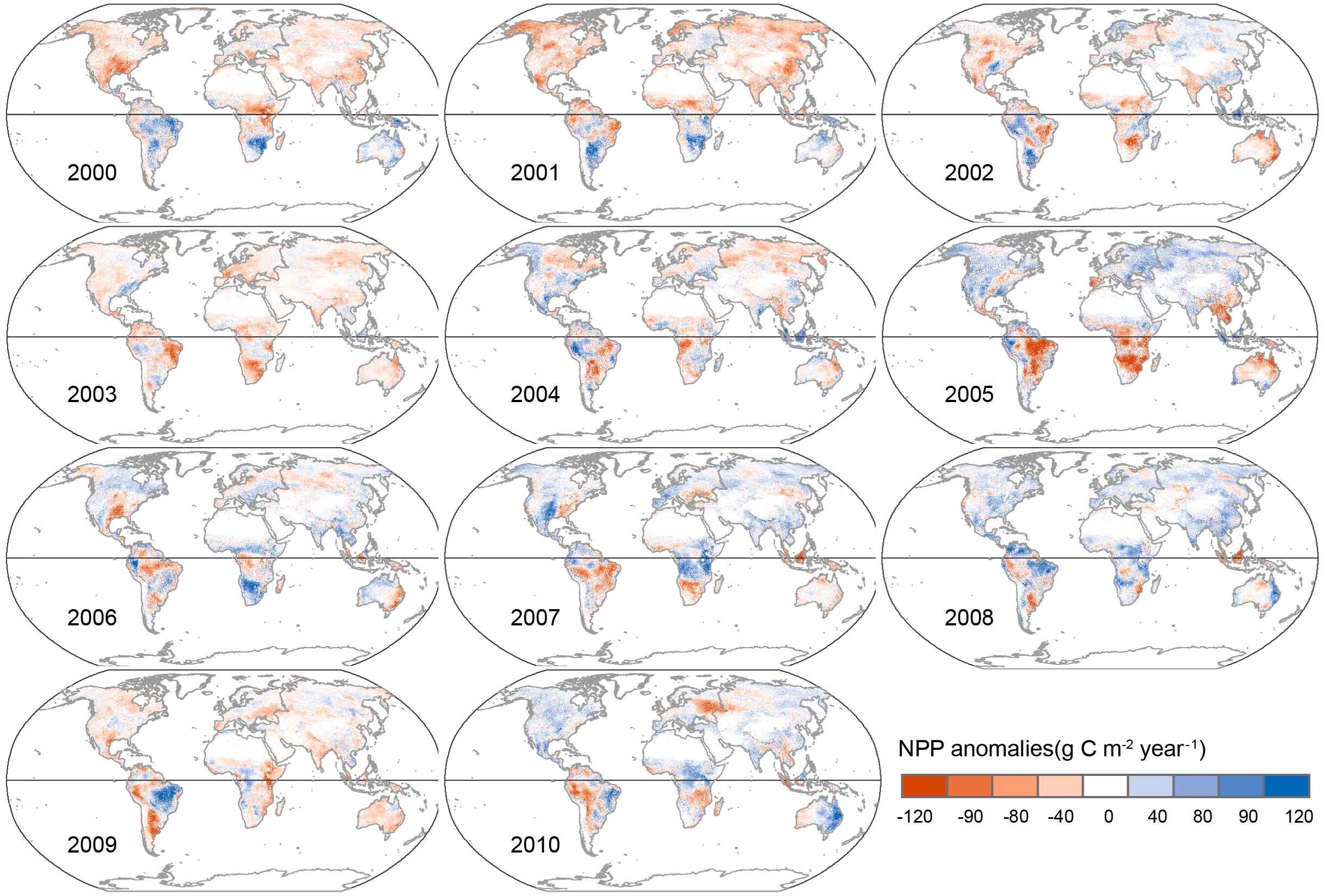


**Supplementary Figure 4. Spatial distributions of terrestrial NPP anomalies from 2000 to 2010.** The NPP anomaly was defined as the difference between the NPP in a specific year and the mean NPP over the period 2000–2010.


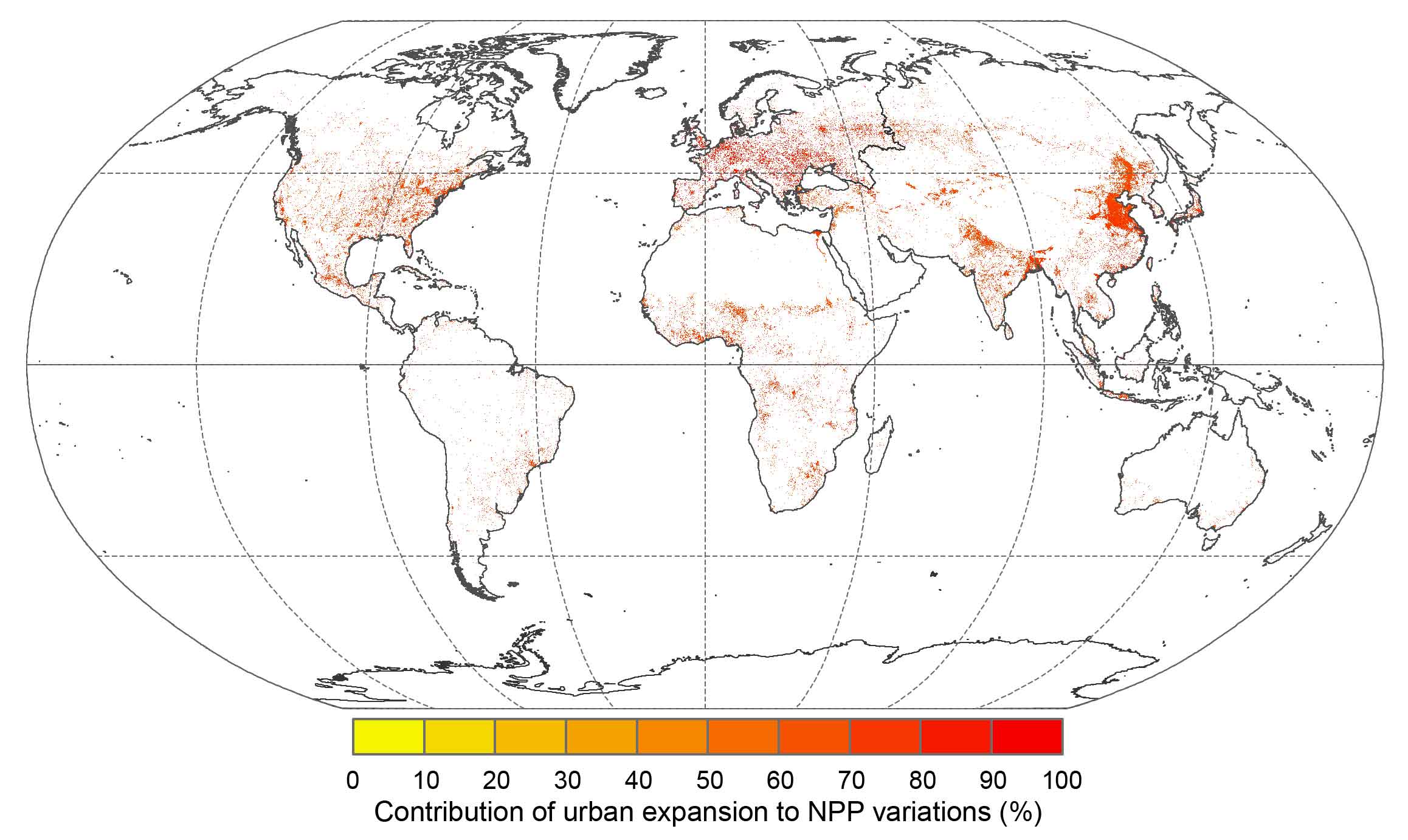


**Supplementary Figure 5. The same as the Supplementary Figure 3, but for the contribution of urban expansion.**


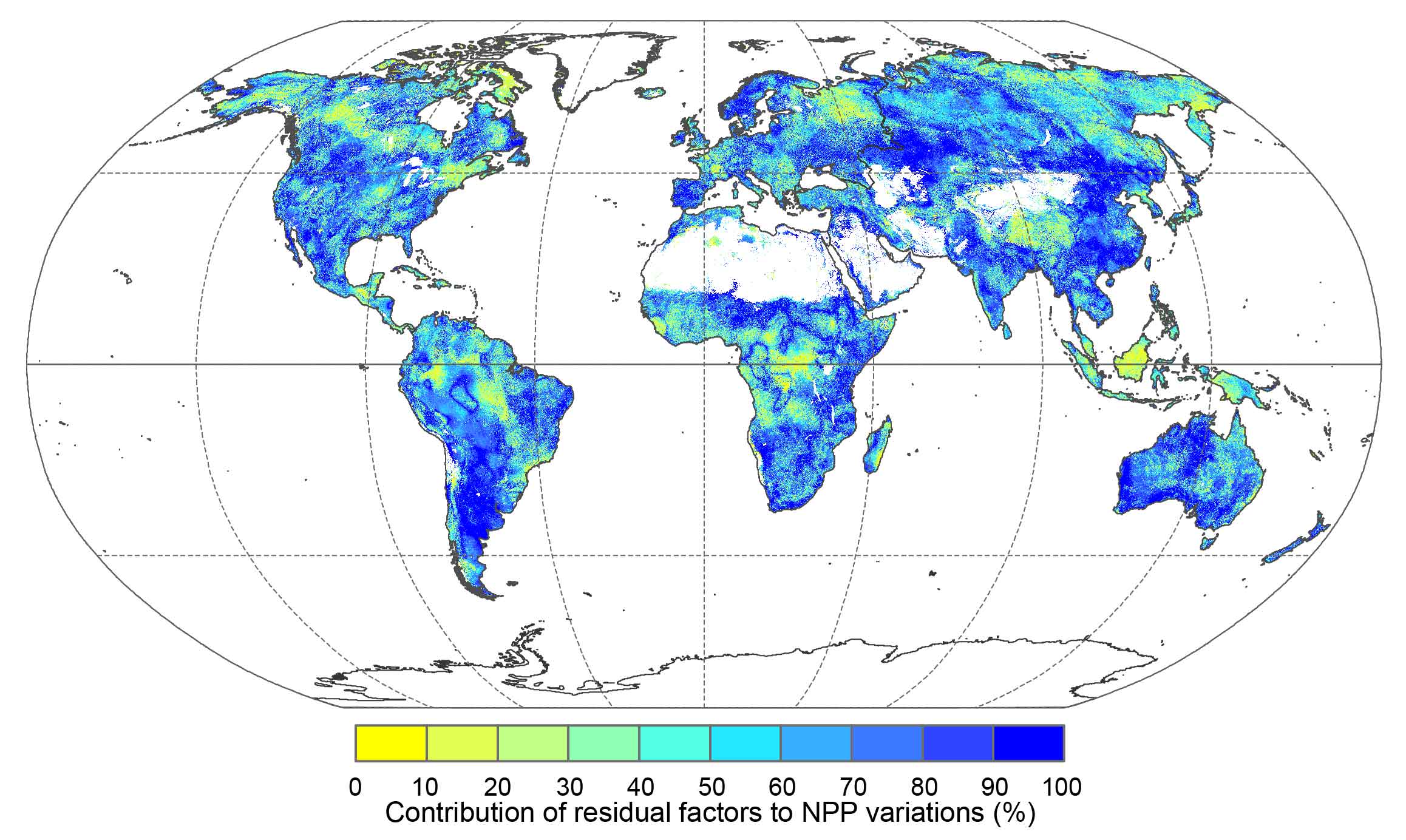


**Supplementary Figure 6. The same as the Supplementary Figure 3, but for the contribution of residual factor unexplained by both climate variability and urban expansion.**





**Supplementary Figure 7. The negative correlation between global terrestrial NPP and carbon dioxide (CO_2_) growth rate from 2000 to 2010 (R=-0.143; N=11; P=0.674)**.

**
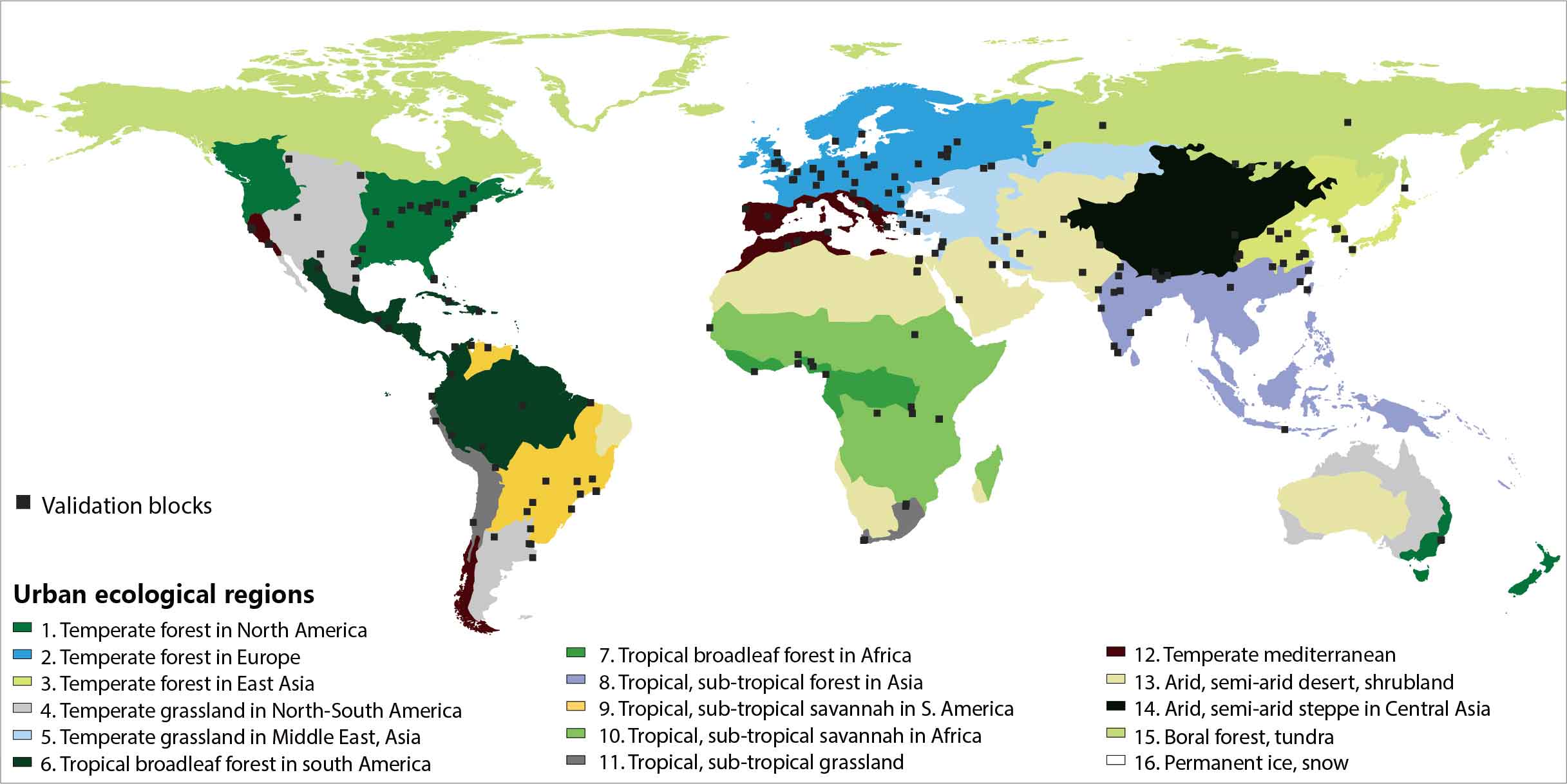
**

**Supplementary Figure 8. The locations of reference data blocks in fifteen urban ecoregions.**


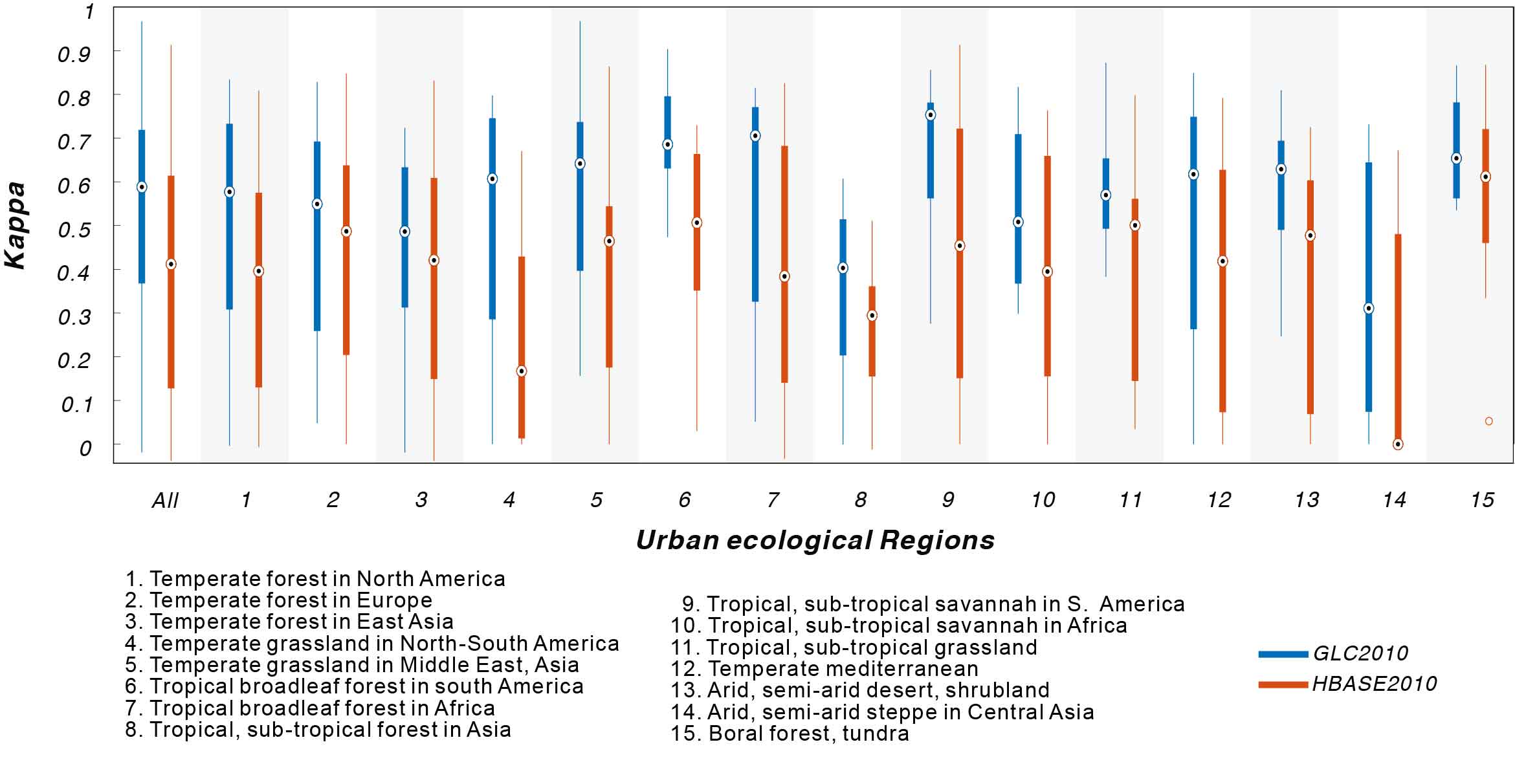


**Supplementary Figure 9. The box-plot of the kappa coefficient for fifteen urban ecoregions.**





**Supplementary Figure 10. Relationships between simulated NPP results from both the CASA and the LPJ-Hydrology, and that from the corrected GPPDI dataset.**

**
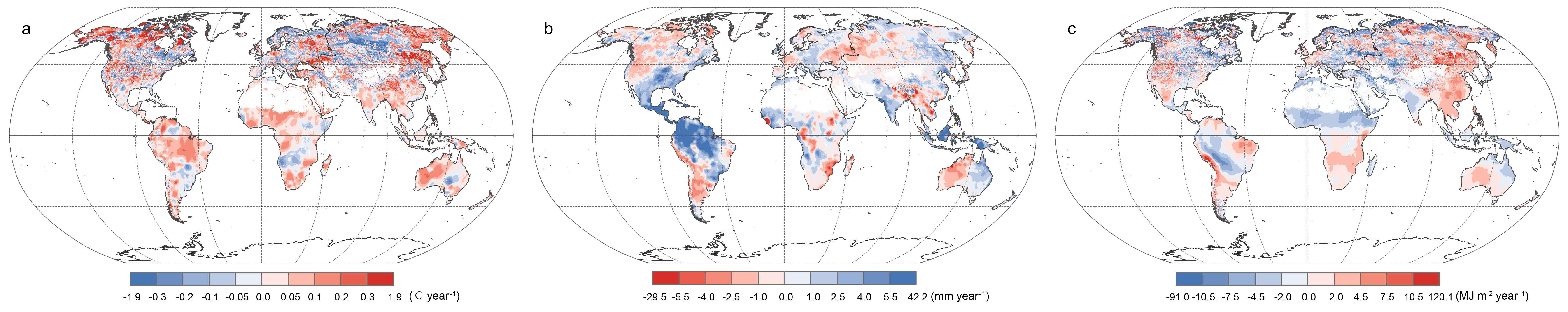
Supplementary Figure 11. Spatiotemporal trends of temperature (panel a), precipitation (panel b) and solar radiation (panel c) from 2000 to 2010**


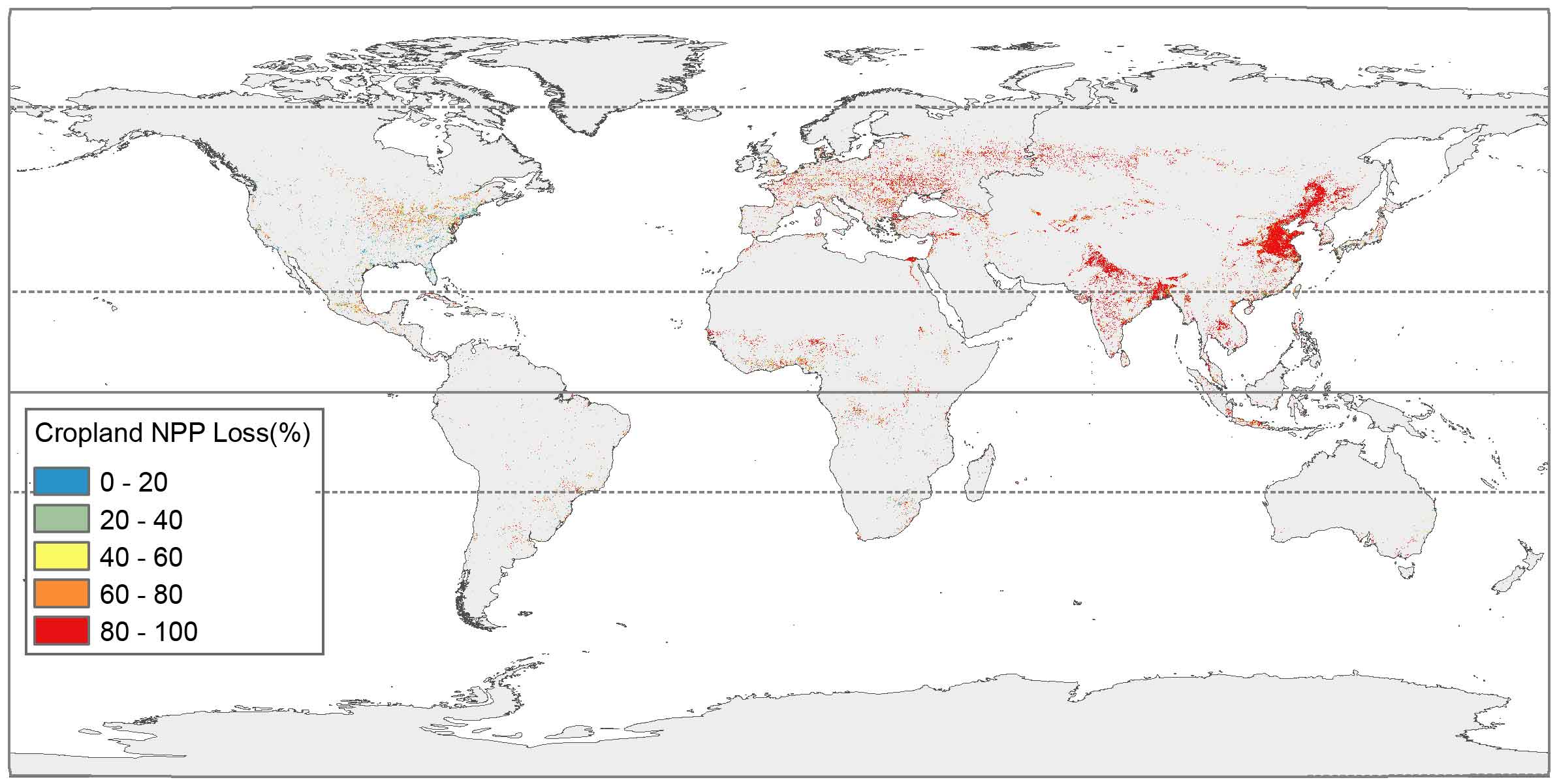


**Supplementary Figure 12. The proportion of cropland-converted NPP loss in new urban lands during 2000–2010.**





**Supplementary Figure 13. Relationship between cropland NPP and grain yield (R=0.818; N=232; P=0.000)**.

# Supplementary Tables

# Supplementary Table 1. Country-based comparison of urban growth and cropland loss from 2000 to 2010.

| Country | Urban area in 2000  (10^3^ km^2^) | Urban expansion area  (10^3^ km^2^) | Urban expansion from cropland  (10^3^ km^2^) | NPP loss due to urban expansion  (Tg C) | Cropland NPP loss from Urban expansion  (Tg C) |
| --- | --- | --- | --- | --- | --- |
| Afghanistan | 1.021 | 0.040 | 0.004 | 0.075 | 0.028 |
| Algeria | 1.500 | 0.252 | 0.048 | 0.560 | 0.196 |
| Angola | 0.857 | 0.846 | 0.044 | 2.371 | 0.234 |
| Argentina | 11.110 | 0.546 | 0.146 | 2.420 | 0.756 |
| Australia | 14.869 | 0.845 | 0.154 | 2.688 | 0.601 |
| Austria | 3.728 | 0.042 | 0.022 | 0.224 | 0.113 |
| Azerbaijan | 2.523 | 0.022 | 0.017 | 0.685 | 0.466 |
| Belize | 0.150 | 0.019 | 0.013 | 0.066 | 0.030 |
| Benin | 0.186 | 0.079 | 0.026 | 0.392 | 0.139 |
| Bolivia | 1.356 | 0.162 | 0.013 | 0.330 | 0.066 |
| Bosnia & Herzegovina | 0.903 | 0.024 | 0.018 | 0.058 | 0.038 |
| Botswana | 1.180 | 0.153 | 0.001 | 0.518 | 0.022 |
| Brazil | 27.405 | 0.573 | 0.146 | 9.464 | 2.666 |
| Burkina Faso | 0.506 | 0.089 | 0.025 | 0.298 | 0.120 |
| Cambodia | 1.157 | 0.038 | 0.029 | 0.403 | 0.353 |
| Cameroon | 0.418 | 0.075 | 0.019 | 1.186 | 0.274 |
| Canada | 20.124 | 1.203 | 0.446 | 5.312 | 1.603 |
| Central African Republic | 0.076 | 0.060 | 0.009 | 0.562 | 0.086 |
| Chad | 0.454 | 0.170 | 0.036 | 0.749 | 0.233 |
| Chile | 2.609 | 0.414 | 0.074 | 0.621 | 0.144 |
| China | 144.061 | 16.053 | 10.899 | 40.561 | 31.412 |
| Colombia | 1.987 | 0.062 | 0.016 | 0.606 | 0.164 |
| Congo | 0.161 | 0.135 | 0.017 | 0.326 | 0.052 |
| Congo, DRC | 1.016 | 0.533 | 0.162 | 4.902 | 1.557 |
| Cote d'Ivoire | 0.484 | 0.300 | 0.070 | 1.921 | 0.498 |
| Croatia | 1.767 | 0.021 | 0.013 | 0.094 | 0.059 |
| Cyprus | 0.228 | 0.033 | 0.014 | 0.045 | 0.023 |
| Czech Republic | 5.174 | 0.041 | 0.028 | 0.281 | 0.167 |
| Ecuador | 1.136 | 0.054 | 0.017 | 0.283 | 0.070 |
| Egypt | 3.611 | 0.486 | 0.248 | 2.179 | 1.596 |
| Ethiopia | 0.581 | 0.208 | 0.079 | 0.985 | 0.448 |
| France | 27.838 | 0.369 | 0.281 | 1.505 | 0.944 |
| Georgia | 0.422 | 0.051 | 0.041 | 0.376 | 0.281 |
| Ghana | 0.625 | 0.707 | 0.313 | 2.170 | 0.948 |
| Greece | 3.780 | 0.087 | 0.043 | 0.210 | 0.103 |
| Guinea | 0.419 | 0.075 | 0.004 | 0.596 | 0.086 |
| India | 48.385 | 0.910 | 0.626 | 10.949 | 8.883 |
| Indonesia | 4.578 | 0.801 | 0.499 | 4.035 | 2.403 |
| Iran | 9.072 | 0.295 | 0.048 | 0.822 | 0.335 |
| Iraq | 2.905 | 0.024 | 0.003 | 0.186 | 0.067 |
| Ireland | 1.135 | 0.102 | 0.017 | 0.065 | 0.020 |
| Italy | 16.617 | 0.285 | 0.149 | 1.002 | 0.459 |
| Japan | 24.462 | 0.392 | 0.095 | 2.828 | 1.153 |
| Kazakhstan | 10.739 | 1.209 | 0.402 | 1.691 | 1.099 |
| Kenya | 0.293 | 0.066 | 0.010 | 0.513 | 0.176 |
| Kuwait | 0.620 | 0.042 | 0.000 | 0.016 | 0.000 |
| Kyrgyzstan | 2.174 | 0.057 | 0.021 | 0.289 | 0.151 |
| Laos | 0.118 | 0.025 | 0.011 | 0.067 | 0.038 |
| Liberia | 0.059 | 0.048 | 0.007 | 0.370 | 0.046 |
| Libya | 1.693 | 0.317 | 0.034 | 0.377 | 0.132 |
| Malawi | 0.682 | 0.022 | 0.001 | 0.379 | 0.059 |
| Malaysia | 1.701 | 0.234 | 0.143 | 1.233 | 0.637 |
| Mali | 0.821 | 0.157 | 0.043 | 0.683 | 0.301 |
| Mexico | 23.195 | 1.806 | 0.784 | 5.664 | 2.218 |
| Mongolia | 0.554 | 0.103 | 0.006 | 0.084 | 0.010 |
| Morocco | 1.926 | 0.142 | 0.032 | 0.683 | 0.260 |
| Mozambique | 1.836 | 0.142 | 0.009 | 1.008 | 0.117 |
| Netherlands | 3.470 | 0.062 | 0.031 | 0.216 | 0.084 |
| Niger | 0.861 | 0.040 | 0.000 | 0.419 | 0.004 |
| Nigeria | 3.700 | 1.083 | 0.426 | 5.647 | 2.296 |
| Norway | 2.147 | 0.247 | 0.007 | 0.163 | 0.008 |
| Oman | 0.872 | 0.159 | 0.003 | 0.032 | 0.007 |
| Pakistan | 6.188 | 0.081 | 0.030 | 1.085 | 0.879 |
| Peru | 1.928 | 0.231 | 0.028 | 0.507 | 0.092 |
| Philippines | 2.399 | 0.058 | 0.036 | 0.497 | 0.369 |
| Poland | 13.139 | 0.616 | 0.474 | 0.651 | 0.378 |
| Portugal | 2.073 | 0.168 | 0.039 | 0.310 | 0.109 |
| Russia | 86.054 | 3.303 | 2.260 | 11.021 | 7.315 |
| Rwanda | 0.051 | 0.026 | 0.017 | 0.105 | 0.074 |
| Saudi Arabia | 6.387 | 0.500 | 0.002 | 0.156 | 0.005 |
| Senegal | 0.948 | 0.150 | 0.047 | 0.647 | 0.335 |
| Serbia | 3.145 | 0.041 | 0.035 | 0.223 | 0.159 |
| Sierra Leone | 0.078 | 0.039 | 0.004 | 0.209 | 0.026 |
| Slovenia | 0.713 | 0.028 | 0.017 | 0.047 | 0.027 |
| Somalia | 0.121 | 0.056 | 0.004 | 0.097 | 0.018 |
| South Africa | 12.765 | 0.751 | 0.089 | 5.653 | 1.293 |
| South Korea | 3.378 | 0.030 | 0.011 | 0.441 | 0.246 |
| Sudan | 4.040 | 0.543 | 0.052 | 1.638 | 0.343 |
| Sweden | 5.592 | 0.072 | 0.012 | 0.284 | 0.051 |
| Switzerland | 1.864 | 0.211 | 0.118 | 0.213 | 0.104 |
| Syria | 3.027 | 0.039 | 0.013 | 0.442 | 0.280 |
| Tanzania | 0.692 | 0.135 | 0.026 | 1.235 | 0.245 |
| Thailand | 3.623 | 0.314 | 0.226 | 1.482 | 1.183 |
| The Bahamas | 0.235 | 0.026 | 0.008 | 0.044 | 0.010 |
| The Gambia | 0.173 | 0.028 | 0.017 | 0.115 | 0.071 |
| Togo | 0.105 | 0.040 | 0.021 | 0.263 | 0.130 |
| Trinidad & Tobago | 0.214 | 0.041 | 0.022 | 0.101 | 0.041 |
| Tunisia | 1.033 | 0.091 | 0.018 | 0.315 | 0.112 |
| Turkey | 9.406 | 0.392 | 0.160 | 1.739 | 1.028 |
| Turkmenistan | 2.690 | 0.028 | 0.006 | 0.358 | 0.204 |
| Uganda | 0.179 | 0.026 | 0.020 | 0.344 | 0.234 |
| United Arab Emirates | 1.083 | 0.174 | 0.000 | 0.017 | 0.001 |
| United Kingdom | 15.352 | 0.405 | 0.232 | 0.805 | 0.372 |
| United States | 220.169 | 11.773 | 4.037 | 47.513 | 13.256 |
| Uruguay | 0.789 | 0.038 | 0.012 | 0.222 | 0.076 |
| Uzbekistan | 11.274 | 0.542 | 0.333 | 1.881 | 1.272 |
| Venezuela | 2.863 | 0.295 | 0.089 | 1.007 | 0.373 |
| Vietnam | 5.451 | 0.686 | 0.516 | 1.709 | 1.244 |
| Zambia | 1.295 | 0.102 | 0.007 | 0.943 | 0.079 |
| Zimbabwe | 1.145 | 0.057 | 0.001 | 0.285 | 0.012 |

**Supplementary Table 2. Maximum light use efficiency of several typical land use/cover types.**

|  | Land use/cover Type | Heinsch et al. 2003  (g C MJ^-1^) | Zhu et al. 2006  (g C MJ^-1^) | This study  (g C MJ^-1^) |
| --- | --- | --- | --- | --- |
| 1 | ENF | 1.008 | 0.389 | 0.607 |
| 2 | EBF | 1.159 | 0.985 | 0.660 |
| 3 | DNF | 1.103 | 0.485 | 0.629 |
| 4 | DBF | 1.044 | 0.692 | 0.652 |
| 5 | MF | 1.116 | 0.475/0.768 | 0.646 |
| 6 | Shrub | 0.774/0.888 | 0.429 | 0.571 |
| 7 | Savanna | 0.768 |  | 0.588 |
| 8 | Grassland | 0.680 | 0.542 | 0.501 |
| 9 | Others | 0.680 | 0.542 | 0.389 |

Note: ENF, evergreen needleleaf forest; EBF, evergreen broadleaf forest; DNF, deciduous needleleaf forest; DBF, deciduous broadleaf forest; MF, mixed forest.

**Supplementary Table 3. Correlations between estimated actual terrestrial NPP and climatic variables, including monthly mean temperature (TAVG), total precipitation (PRCP) and downward shortwave solar radiation (SRAD)** over both hemispheres and the entire globe from 2000–2010.

|  | COR(NPP, TAVG) | COR(NPP, PRCP) | COR(NPP, SRAD) |
| --- | --- | --- | --- |
| Northern Hemisphere | 0.910^**^ | 0.815^**^ | 0.973^**^ |
| Southern Hemisphere | 0. 984^**^ | 0.896^**^ | 0.879^*^ |
| Globe | 0. 882^**^ | 0.692^**^ | 0.973^**^ |

^**^significance level of P < 0.01

^*^ significance level of P < 0.05

**Supplementary Table 4. Datasets** used in this study.

|  | Dataset | Temporal Resolution | Spatial Resolution | Sources |
| --- | --- | --- | --- | --- |
| 1 | Land use /cover | Yearly | 30 m | GlobeLand30, <http://www.globallandcover.com/GLC30Download/index.aspx> |
| 2 | Land use /cover | Yearly | 500 m | MCD12Q1, <https://lpdaac.usgs.gov/data/get-started-data/collection-overview/> |
| 3 | Gridded NPP data | Yearly | 1 km | MOD17A3, <https://lpdaac.usgs.gov/data/get-started-data/collection-overview/> |
| 4 | NDVI | Monthly | 1 km | MOD13A3, <https://lpdaac.usgs.gov/data/get-started-data/collection-overview/> |
| 5 | Temperature | Monthly | 0.5° | [http://www.esrl.noaa.gov/psd/data/gridded/data.ghcncams.html](http://www.esrl.noaa.gov/psd/data/gridded/data.ghcncams.html ) |
| 6 | Precipitation | Monthly | 0.5° | <http://www.esrl.noaa.gov/psd/data/gridded/data.gpcc.html> |
| 7 | Wet-day Frequency | Monthly | 0.5° | <https://crudata.uea.ac.uk/cru/data/hrg/cru_ts_3.24.01/> |
| 8 | Solar Radiation | Monthly | 0.5° | <http://hydrology.princeton.edu/data.php> |
| 9 | Cloud Cover | Monthly | 0.5° | <https://crudata.uea.ac.uk/cru/data/hrg/cru_ts_3.24.01/> |
| 10 | Soil data |  | 30 arc-second | <http://www.fao.org/soils-portal/soil-survey/soil-maps-and-databases/harmonized-world-soil-database-v12/en/> |
| 11 | Field-measured NPP |  |  | Global Primary Production Data Initiative Products, [http://daac.ornl.gov//NPP/guides/NPP_GPPDI.html](http://daac.ornl.gov/NPP/guides/NPP_GPPDI.html) |
| 12 | CO_2_ data | Yearly |  | CO_2_ data, <https://www.esrl.noaa.gov/gmd/ccgg/trends> |
| 13 | Grain yield | Yearly |  | Statistical Yearbooks, <http://tongji.cnki.net/kns55> |

# Supplementary Notes

**Supplementary Note 1: Impacts of global urban expansion on terrestrial NPP and crop production**

To examine the effects of urban expansion on terrestrial NPP, the NPP losses induced by urban land growth were analyzed at a continental and national scale. Our results showed that Asia experienced considerable NPP losses due to urban expansion between 2000 and 2010 (87.3 Tg C; Fig. 4). In particular, the NPP losses in China (40.6 Tg C) accounted for 47% of the NPP losses across the entire continent, and 18% of the total NPP losses from global urban expansion during the same period (Supplementary Tab. 4). The NPP losses in China were comparable to those in the United States (47.5 Tg C), and more than twice the NPP loss occurring in Europe (20.2 Tg C). Despite relatively small urban expansion in South America and Africa, both experienced large reductions in NPP (Fig. 4). This was associated with the higher density of pre-urban NPP in South America and Africa (694 g C m^-2^ year^-1^ and 548 g C m^-2^ year^-1^, respectively), in comparison with other continents.

In addition, we found that more than 70% of the NPP reduction in the newly urbanized areas in Asia was due to cropland losses and in East Asia, this reached approximately 85% (Supplementary Figure 12). The area of urban expansion, cropland conversion, and their corresponding NPP losses were analyzed for the 100 largest countries worldwide by continent (Supplementary Table 4). A significant linear relationship was found between urban expansion area and the corresponding NPP losses (R=0.953; N=100; P=0.000). Furthermore, a strong correlation was also found between urban-expansion-based NPP losses and the cropland-driven NPP loss (R=0.888; N=100; P*=*0.000). The city-based analysis also revealed a significant correlation between cropland-driven NPP loss and grain production (R=0.818; N=232; P=0.000) (Supplementary Figure 13). This is consistent with the findings of Lobell et al. ^69^ and indicates that urban expansion could threaten the terrestrial NPP and crop production worldwide.

**Supplementary References**

1. Lobell, D.B., et al.. Satellite estimates of productivity and light use efficiency in United States agriculture, 1982–98. Global Change Biol 8, 722-735 (2002).
